# Ethics and etiquette in an emergency vaccine trial. The orchestration of compliance

**DOI:** 10.1080/11287462.2020.1726591

**Published:** 2020-02-21

**Authors:** Arsenii Alenichev

**Affiliations:** a Department of Anthropology, University of Amsterdam, Amsterdam, Netherlands; b Barcelona Institute for Global Health, Barcelona, Spain

**Keywords:** Ebola, clinical trials, bioethics, bioetiquette, compliance

## Abstract

Participant non-compliance and withdrawal from randomized clinical trials has increased focus on analysing the results from the “per-protocol” population that complies with a trial’s protocols. There is no clear understanding of what shapes protocol compliance in practice. In this paper, I theorize clinical research from the perspective of participants in an Ebola vaccine trial by analysing the practices that contributed to very high compliance rates. In this setting, per-protocol compliance became an essential component in forming a class of “proper” researchers and participants working together in the rapidly expanding market of clinical research. Bioethics supports participants’ right to withdraw from research as an ethical safeguard in the process. But participants seeking affiliations with powerful institutions may voluntarily embrace their trial responsibilities over a right to withdraw. To understand this phenomenon, this analysis uses the notion of *bioetiquette* – the set of rules specifying “proper” and “improper” trial subjects and behaviours – which runs in the shadow of formal bioethics in trials and requires careful transdisciplinary examination.

## Introduction

In 2014, I trained to become a bioethicist at Case Western Reserve University (Ohio, US), with a specialization in international research ethics. During my training, I learned several key premises. First, bioethics is a multidisciplinary and sometimes vague field that explores what is right and wrong in science, medicine, and beyond, and it aims to protect “vulnerable” people from exploitation by medical and research institutions. Second, there was a significant number of unethical studies in Europe and the United States in the twentieth century that became widely known after the research occurred. Third, when experiments and transnational institutions travel to the “global south”, exploitation, coercion, and undue inducement of vulnerable participants can happen. “Parachute” studies, when research teams simply drop in to a location to collect samples and then fly back, are ethically problematic. Fourth, informed consent procedures, institutional review boards, and the responsibilities of researchers are the key safeguards to ensure that global health trials are ethical. Fifth, even though there is no universal way to make trials ethical, tailoring international guidelines to local contexts along with increasing community engagement and collaborative partnerships are important elements for good international research.

After graduating, while working as a clinical research associate, I learned that the compliance of people enrolled in clinical studies is of critical importance for researchers and sponsors for two main reasons. First, it is easier to run statistical analysis when participants follow all of the planned procedures without deviations. Second, research institutions that can run trials with high compliance rates are likely to attract other projects. Identifying a per-protocol population allows researchers to present high-quality data to sponsors, and potentially helps a trial advance from an experimental phase to a licensed product on the market. Thus, an ability to execute high-compliance trials is an important performance indicator amongst research units competing for projects.

In an ideal scenario, every subject enrolled in a clinical trial would voluntarily and consciously follow the set protocol, pursue this until completion of the trial, and thus contribute data that were complete in all respects (Ranganathan, Pramesh, & Aggarwal, 2016). Clinical researchers distinguish two types of study populations: the “intention to treat” group, which includes all participants who enrolled in the trial, and the “per-protocol” group, or those who completed the study without major protocol deviations (Sanchez & Chen, 2006; Yellowlees, 2017). To encourage per-protocol compliance and keep “subjects on protocols” (Lopienski, 2015), research teams may adopt certain ethically permissible techniques, such as expressing friendliness and care for participants; dispelling myths, misconceptions, and rumours about trials; and providing participants with some compensation or gifts. Formal bioethics assumes that participants will act autonomously and use their right to withdraw from the trial at any point should they desire to. High compliance rates are therefore considered a sign by many researchers that participants gave voluntary and informed consent to participate in research, and that researchers provided information about participation in a careful and sensitive way.

But how does the making of per-protocol compliance happen in practice? How do “healthy” participants relate to research benefits? Or, more precisely, why do people decide to comply with study procedures? My training allowed me to enrol in an anthropology PhD programme and study bioethics in practice in an Ebola vaccine trial in West Africa. In the context of both a humanitarian and a scientific emergency, the Ebola vaccine trial flawlessly followed the ethics procedures I had been taught, including establishing collaborative partnerships, promoting community engagement and culturally sensitive interventions. To achieve this the research team, locally dubbed “Little America”, coordinated the work of more than two hundred staff members who provided information to participants, and where needed, proposed adjustments. The trial staff trained a special group of mediators – the trackers – local people from the communities where the study was taking place, whose task it was to “track” participants, thereby facilitating compliance. Between 2014 and 2016, 1,500 participants joined the trial; at 98 percent compliant, they were an almost ideal per-protocol population.

In October 2016 I began to conduct qualitative research in a community that hosted the Ebola vaccine trial, working with volunteers who had recently completed their participation in the trial. I use pseudonyms here to ensure their anonymity. All of those quoted in this article were legal adults. In accordance with guidance from the ethics board of the trial institute, I provided them with US$2.50 as compensation for their participation in interviews and focus group discussions. In this residential area, where poverty, unemployment, and lack of food and healthcare were common, this was not an insignificant sum. My research assistant referred to the area as a “slum”, and my informants often described it as a network of “bases”, meaning areas inhabited by alienated youth and ex-combatants of the still recent civil war. Most of my informants were illiterate and underemployed. They explained that they received about US$300 for participating in the trial, and that US$70 was the average monthly wage for people in that area. Their compensation was provided to them at different moments of the trial: US$40 for the vaccination visit, US$10 for each regular follow-up visit, and US$150 at the “graduation” – a ceremony for subjects who successfully completed the trial. Informants further explained that they got small packages of food from the researchers, and had the chance to receive better healthcare. Some were disappointed that the ID cards – issued upon recruitment into the study – were taken away after graduation. Participants showed me their trial certificates, which strikingly resembled my own diploma in bioethics.

**Figure:**
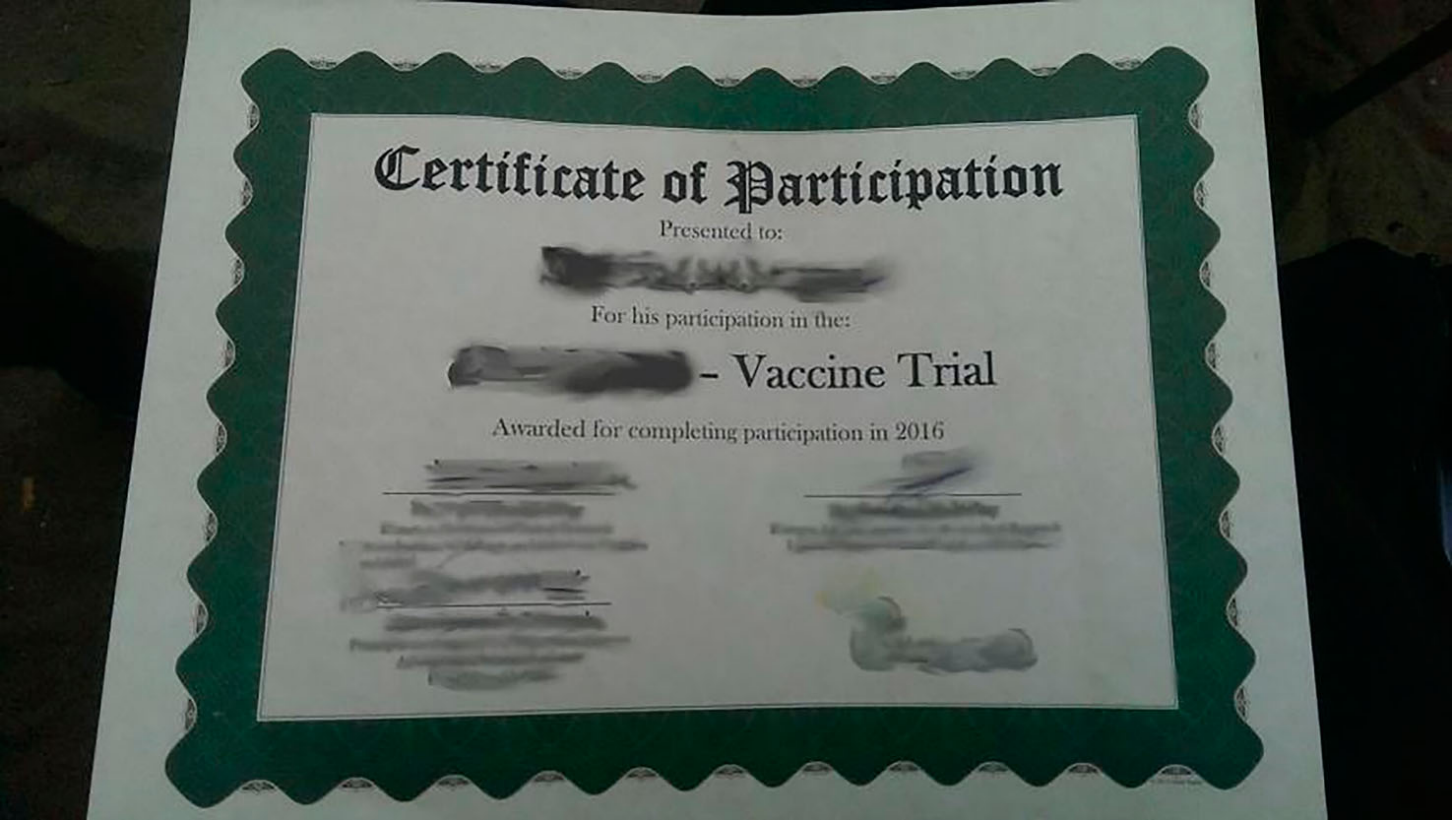

During my research, I encountered a situation that many critical bioethicists and anthropologists have expressed concerns about: “healthy” clinical trial participants often approach their participation in research as a kind of precarious employment, beyond altruistic volunteerism, something that highlights the awkwardness of the cheerful formal bioethical discourse of “gifts” and “compensation” (Abadie, 2015; Bernstein, 2003; Fisher, 2013; Lemmens & Elliott, 2001; Zvonareva et al., 2019). Others have proposed the notion of *clinical labour* to conceptualize the participation of healthy volunteers in trials (Cooper & Waldby, 2014). Indeed, real world moralities are far more complex than authoritative codes and guides suggest (Mattingly & Throop, 2018; Zigon & Throop, 2014). In this article, I contribute to the scholarship on this issue. Building on earlier studies, I will argue that, like any other kind of labour, clinical labour has its own etiquette: trial subjects adjust their behaviours to the requirements of the trial in attempting to deliver per-protocol compliance. Adapting Martin and Stent’s (1998) term *bioetiquette* to the trial context, I propose that we rethink prior ways of using it.

## From Little America with love

As I started interviewing researchers who had launched the Ebola vaccine trial, I learned that for many it was a kind of competition: to become the first to test a vaccine that could save West Africa and the whole world from Ebola, and, equally importantly, to establish the first large-scale research unit in the country. By 2016, the researchers had been able to complete several successful Ebola-related projects, and the research unit itself was recognized as a local pioneer of human subject clinical research. By 2018 the unit had begun preparing for HIV and malaria research, indicating that research goals had shifted from Ebola to other diseases common in the region. In an era when state sovereignty is being exceeded by proliferating international organizations that are independently addressing issues in healthcare and development, Little America enacted a “mobile sovereignty” that produced a new kind of citizenship for people participating in biomedical interventions (Nguyen, 2010). As a trial participant explained, this citizenship had its perks:

If we are sick and we visit the hospital they would take us off the line and treat us [before the others]. We literally had our own clinic in the hospital. (Dulee)

During a tour of the research unit, I observed the wealth poured into the facility, with its tap water, air conditioning, conference rooms, top-level medical equipment, vehicles, disposable hygiene items, and Wi-Fi: all luxuries in the region, typically limited to some of the most prosperous NGO offices, governmental facilities, and expensive hotels. No wonder the staff called the research unit “Little America” – a nickname that contrasted starkly with that of the crowded, resource-poor local hospital that was known locally as “Just for Killing”. Staff at the research facility often wore colourful, short-sleeved polos printed with team logos, and some of them frequently travelled to the United States and Europe for presentations and training. The facilities included a “consent room”, located among doctors’ offices, where trial educators spoke with potential participants about the trial’s procedures and obtained their consent to participate. This area reminded me of a production line: people moved from one room to another, organized and efficient, civil and friendly.

## Chasing Little America with good behaviour

However, just as Little America contrasted with the rest of the hospital, their neat graduation diplomas contrasted with the precarity in which my informants lived. Among those I interviewed, participating in research was a hopeful attempt to be included in the rapidly developing research sector being powered by Little America, with all the medical, social, and economic benefits it bestowed. From trial documents and conversations with trial volunteers, I also learned about a plethora of rules for being a “good participant”. Trial subjects regularly went to the research unit for scheduled appointments, expected home visits, and adjusted their lifestyles to meet the trial’s expectations. When acting in accordance with all these rules, volunteers received payments, healthcare and food packages, which enabled them to facilitate affiliation with a research unit, while keeping hopes of graduation and awards in the future. Importantly, Ebola was seen as a shameful situation, and the trial and its good participants were seen as working collectively to prevent the country from Ebola-related shame. Sando, one of the trial graduates, explained:

[Researchers] said that the vaccine was coming into the country to help to protect us from future **embarrassment** of Ebola. (Sando’s own emphasis added^1^)

Along with the desire to avoid embarrassment, informants explained that they wanted to support and accelerate the trial by inviting people from their social circles to join in. Hence, they advocated for participation, in a similar manner to professional community engagement teams.

## Getting started

Informants told me that it was relatively difficult to get in the line to obtain a ticket to enter the research unit because so many people wanted to participate. Some awoke as early as five in the morning to stand in line, sometimes several times, to secure a spot in the workshop about trial participation. This meant that participants had to adjust their daily routine – employment, child care, transportation – to be admitted to the research unit. Informants further explained that researchers had an appointment sheet, and that people were put on a long waiting list, planned for three days ahead, as Dioh explained:

*[*Researchers] listed appointment sheet and they give it to each person for three days [in advance]. After three days we went there … To participate you have to wake up early. I tell you: my man, go over there [to the hospital] at five thirty in the morning. (Dioh)

In workshops participants were advised to tell the truth to researchers; the trial participant brochure read: *“*It is important for you to say the real truth so the study team can make sure it is safe for you to be in the study.” Trial brochures also warned that people should minimize blood and body fluid exposure after the vaccination. Specifically brochures gave polite instructions to avoid open-mouth kissing, as well as sharing needles, razors, forks, spoons, cups, toothbrushes and other items. People were instructed to avoid pregnancy, and to use condoms during any form of sex for two months after the vaccination. In accordance with trial inclusion criteria, pregnant and breastfeeding women were excluded, and women with childbearing potential were required to take a urine pregnancy test. Trial subjects also needed to be a certain healthy body size to be included in the trial, and that generated shameful situations:

When they first offered the vaccine, it was not for sick person. Even if you're not sick and you go there [there is a possibility] you wouldn't get the vaccine. They will drop you from the group [of potential participants] … They made it on one little boy in my presence, due to his condition. He was very small in his body. **He was a tight man but he was very small in his body, they [researchers] scaled him and they denied him of taking the vaccine.** (Moses)

## Managing mindset, lifestyle and social emotions

To become trial subjects, participants had to be prepared to be tested for HIV and syphilis, which are two highly stigmatized conditions, with potentially heavy judgements about the participants’ lifestyle. Moreover, their lifestyle had to be adjusted to meet research goals:

Actually after [researchers] take the blood from us, they give us the money, they said **we should buy food** to eat and they told us **not to smoke**. We shouldn’t **do hard work**. We shouldn’t **travel** to go **anywhere**. We should **remain to our place** until the entire programme is over … We shouldn’t **drink alcohol.** (Luogon)

Moses, for instance, informed me that he decided to move closer to the research unit for this purpose: to stick around. For some, the lifestyle adjustment was a challenge. A trial graduate, John, told me that he had adverse effects from the vaccine, and left to go to see the traditional healers outside the city, counter to the researchers’ request to stay near the research unit:

[After the vaccine shot] my whole body was full of kro-kro (itch) … Itch big-big itch. I had to go to the [hinterland] for traditional medicine, and I rubbed it and since that time … **You couldn't go out of town to the rural area until the study is over**. (John)

**Figure:**
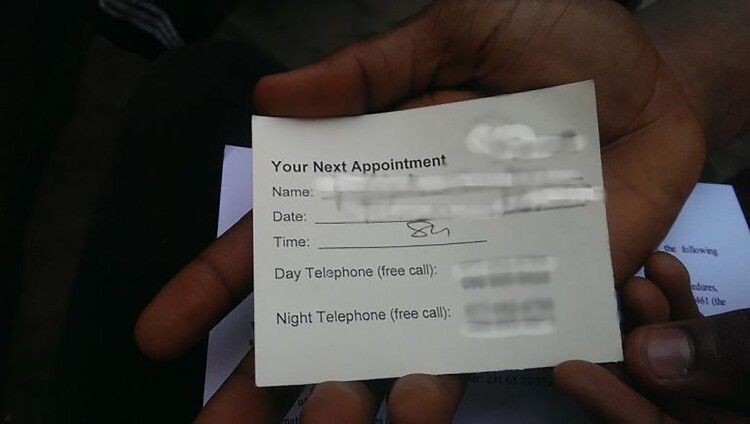

The trial was also accompanied by persistent stigma and rumours. The attendant opprobrium visited on people affiliated with Ebola activities formed an obstacle for numerous people, and dealing with it required emotional intelligence. This meant that per-protocol participants had to be able to overcome stigma-induced negative social emotions, including other people’s fears that they might get Ebola or die because of their participation. Participants had also to deal with the fact that they were often identified as belonging to precarious marginalized groups, so-called *zogos,* that are widely shamed and problematized at the national level. In addition to managing social emotions, trial subjects had to honour biomedical knowledge by accepting its undeniable validity. In doing so, traditional knowledge formations that injections, as acts of skin penetration, can affect bodies, making them strong or sick, pregnant or sterile, had to be dismissed as shameful and embarrassing “rumours” and “misconceptions”. Hence, volunteers had to face family and friends incarnating these perceptions, as well as overcome their own insecurities about the blood drawing and blood collection that were part of the research.

## Maintaining good health

Informants recounted how the research teams told them that they should use their “compensation” money to buy food and vitamins to sustain their body. For example, Momulu told me:

They said the money was to help us to buy one or two stuffs [items of food] for vitamins so we can **regain our previous [health] status again.** (Momulu)

It was not always easy for participants to stay healthy because of the side effects of the vaccine, but also because of the overall scarcity of social, economic, and health resources. Wonbin, a trial participant, explained that trial subjects should have received healthcare to manage adverse reactions to the vaccine, but the hospital could not provide this. If you have a fever or headache, he said,

[they] tell you that you should be going to the hospital for treatment.But the hospital can’t provide treatment. And anytime we go there, they will hand us bad talks.They will act as if we did not voluntarily participate.

Participants were also sometimes expected to buypills with their own money to manage their sickness, as Emmanuel explained to me: “Sometimes they say ‘[the] medicine is finished’, and they will give you a paper to go buy itat the drug store.”

## Investing in relations with trackers

Good participants were expected to be ready for visits by community members who were working for the research teams, so-called trackers. According to informants like Asha, the trackers often scheduled home visits without negotiating the time and date: “It is him who decides [about date and time]. It's him.” Trial participants thus worked to maintain a good relationship with their trackers as a prerequisite to graduate and get paid. Miata, for instance, was afraid that she might not be considered a per-protocol participant: “Trackers had to keep [a] record on you; that was the only way you will receive your money.”

Similarly, Chea was concerned about trackers threatening to exclude people from the study because of the way they participated:

[Trackers] went as far as threatening us, saying**: the way you've already taken this vaccine** [in regards with compliance], in fact we will take you from the study. (Chea)

Some participants complained that researchers had promised to give them mobile phones to communicate with the research teams. In the context of this community, phone communication was generally a problem. Few participants had mobile phones, electricity to charge them, and credits to call. A “good participant”, one might infer, should always be able to take a phone call from trackers, or to phone them, if need be using their trial compensation for this. Gyude clarified:

[Researchers] said if we take the vaccine we will get benefits and, two, we will get [a] phone … That they will share [a] phone among us. They said plenty things. My tracker, you know, for me to tell my tracker how I'm coming on, but they did not share [a] phone with us. **We took our own money and buy phone with it**. (Gyude)

Along with struggling to get and keep a phone, informants expressed a concern that having a phone and having good relations with trackers could influence whether participants would be invited to join in future Little America projects. Henry explained:

My study [next clinical trial for graduates] will be opening [soon], and if you don’t have a phone you will be out of the study. And [researchers] **ask your tracker if you can cause a problem for him, [and if so] they'll take you out of the study.** But the most important thing is you should have phone. Yes, that was what [a tracker] told me, and I told him that I don’t have phone and **he said that I need to get a phone**. **How will we get a phone?** (Henry)

## After graduation: aspirations and anxieties

They said if we were going to be **successful** during the vaccination process, we will benefit in the future. (Jue)

Several paid volunteers hoped to be inv**i**ted to participate in other projects after graduating from the present trial; such opportunities might include another vaccine trial or long-term follow-up research. Being considered a “good participant” was critical to such invitations. Good participants who dutifully adjusted their behaviour to follow the research protocol had better chances of securing benefits and being selected for other projects run by Little America. Tarloh explained that graduates were expecting to get t-shirts from Little America, to signify their belonging, as well as to do advocacy in communities. Some trial participants opined that some graduates should get to go to Big America. Stories circulated among the participants about a girl in Minnesota who was believed to enjoy many benefits from participation. Tamba told me:

I didn’t benefit because they only give me US$150 [for the Graduation], and that is not a benefit compared to what I did. The most important information I heard about the vaccine was the future benefit they promise us, because they told us about **a girl in Minnesota that took the vaccine and she’s still alive with her benefit.** (Tamba)

Moreover, the certificate suggested a long-term reciprocity that was not possible within the realities of standard operational procedures. Many participants felt disappointed by the end of participation: their after-graduation narratives included themes of broken promises, missing benefits, and inability to be affiliated with the research unit to the extent they had been hoping for. Some participants like Joseph complained that their graduation certificates were unlaminated and showed signs of damage caused by humidity, whilst the certificates of others were sealed, shiny, and glossy:

Rotten paper they give us. Rotten, **they did not even put it in plastic**. (Joseph)

In particular, they expressed a concern that stories about participation and the promise of a glossy certificate were used to entice them, and ultimately their participation generated benefits mostly for the government, research teams, and the institutions that sponsored them. Some graduates were frustrated with participation in the trial, how it was carried out and justified, with notions of being cheated and used, so they threatened trackers:

Now we threaten [trackers] on the streets and say: ‘**We will beat y'all and kill y'all and war comes’** because they put stupidity in our bodies, and they don't want to give us nothing. (George)

## Etiquette as an implicit technique

When I juxtapose what I learned as a bioethicist and what I encountered in the field, it is clear that the discourses and practices of bioethics do not map onto each other. Just as my own diploma legitimized me as a skilled bioethicist, informants’ diplomas legitimized them as responsible participants able to act properly in research activities. It is impossible to conceptualize the complex dynamics that I observed on the ground in the terms used in formalist bioethical discourses. Several processes ran simultaneously alongside each other. Researchers adhered to good research protocols and bioethical principles, and facilitated adherence and compliance by incorporating collaborative partnership and engagement practices. Informants internalized and enacted “proper”, “per-protocol” participation behaviours. Participants acted consciously and voluntarily in a specified manner with high hopes of upward mobility, amid fears of being denied benefits, being removed from the trial, or being excluded from participation in subsequent trials. The morality pertaining to desired behaviours of participants and the moral judgements about trackers and researchers suggested that an unarticulated but crucially important etiquette was in place alongside formal rules of ethics.

Social scientists have long argued that etiquette encompasses more than formal rules, but should be understood as a ritualized form of submission to a power hierarchy that organizes social interactions within a particular context. The word “etiquette” comes from the Old French verb *estechier* (also *estichier*, *estequier*; to attach, stick) and was used to indicate the “superior” behaviours of the aristocracy who were privileged to be attached to or near the sovereign. By acting as a technique of recognition and acceptance, etiquette enabled people to enact social hierarchies and to practise their belonging (Elias, 2000). For Elias, the driving force of etiquette was the internalization of shame and embarrassment within the “improper” other (Elias, 2000). Goffman (1956) argued that etiquette is largely about the presentation of the self, and that it is a conventionalized ceremonial code that acts as a means of communication. Etiquette also goes hand-in-hand with social aesthetics (Bovone, 1993; Coleman, 2013). Crapanzano notes that the words “etiquette” and “stigma” share the root word *steig*, and highlights a similarity between stigma and etiquette as social processes that place positive and negative “marks” on people (Crapanzano, 2001).

Etiquette rules have been developed to smooth out many social occasions. In recent years forms of etiquette have significantly proliferated, they became more flowing and flexible, differentiated and varied in their manifestations (Wouters, 2004). Books on business etiquette describe how to treat co-workers in order to maintain an effective, polite, and pleasant work space. There are guides that teach how to politely eat, sleep, talk to other people, and how to modestly urinate and defecate. There are many publications on etiquette related to gift-giving, visiting hospitals, and volunteering (Lovering, 2019; Mayne, 2018). Rules of etiquette are common in major transnational organizations, where they may help maintain a positive image and reputation for the organization. For instance, the World Health Organization has banned smokers from employment (World Health Organization, 2008). The organization has also publicized guidelines on how doctors, especially when they are men, should talk with women about intimate partner violence (World Health Organization, 2013), and has even developed guidelines on how to cough (Granich et al., 1999). It is no wonder then that biomedical research, and its numerous social interactions that are theorized by bioethics, has an etiquette, or perhaps a bioetiquette, of its own.

## Business etiquette for clinical labourers

Within the discipline of bioethics, the participation of healthy people in clinical research is theorized as a voluntary contribution to science and development, despite the overwhelming evidence that healthy trial subjects often participate in trials for financial reasons (Lemmens & Elliott, 2001). Anthropologists have recently introduced the concept of “biovalue” to highlight the body’s valuable ability to sustain itself and, when subjected to research interventions, respond to irritants, such as an injection of experimental vaccines (Birch & Tyfield, 2013). In the light of a growing concern that contemporary labour markets push people into more flexible and semi-regulated kinds of work, economic activities involving the commodification of biovalue have been conceptualized as “clinical labour” (Cooper & Waldby, 2014).

In practice, as a new kind of labour, clinical labour comes with specificities that other kinds of work do not necessarily have, such as stigma and fears about bodily risks. Those context-specific obstacles may lead to participants dropping out and not complying with the protocol, which destabilizes clinical research. As participants learn to comply with and adhere to protocol, a new profession of “trial subject” emerges as the biomedical industry develops techniques for “keeping subjects on protocols” (Lopienski, 2015). People begin to behave and organize their routines in relation to risks, fears, pragmatic concerns, pressure from researchers (do not be afraid of needles, avoid body fluid contacts, get a mobile phone, avoid breastfeeding, minimize issues with trackers, control your body size, avoid travelling, avoid alcohol, avoid hard work) in order to trade their biovalue for socioeconomic benefits.

Theorizing participation in clinical trials as a form of clinical labour raises another analytic point: bioetiquette in clinical trials is a kind of business etiquette for clinical labourers. Attending to this bioetiquette does not depend on any notion of suffering subjects, but casts trial subjects as entrepreneurs with their own trajectories and motivations. While bioethics foregrounds a formal acknowledgement of participants’ rights to withdraw from research, bioetiquette stipulates the responsibilities people must accept in order to become affiliated with powerful organizations. Not only does it involve a persistent moral pressure and a threat of embarrassment, but it acts as a kind of lubricant for social, “per-protocol” relations with biomedical institutions.

## Rethinking research protocols as etiquette guides

It is quite remarkable that one of the definitions of the word “protocol” is a “set of strict rules of etiquette with a goal to maintain adherence and precedence” (Merriam-Webster, 2019). Whilst this meaning is common in diplomatic and military settings, etiquette rules emerging from trial protocols and related documents indicate that this definition might be relevant to biomedical research as well. Based on trial-related documents and data collected from my informants in marginalized areas, it is possible to reconstruct some elements of the bioetiquette that was at work in this specific research context:

- You have to wake up early to get an appointment ticket and adjust your routine accordingly
- As a participant, you should not smoke, drink alcohol or do hard work
- You should remain at your place, avoid travelling or even move closer to the research unit so you are easy to reach by the research team
- You should not be afraid of HIV and syphilis tests, pregnancy tests and lifestyle judgements
- You should avoid body fluid contacts and adjust your social and sexual lifestyle accordingly
- You should not be afraid of injections and not have suppositions about them
- You should not be afraid of stigma and you should dispel misconceptions
- You should invite more people to join the study, e.g. your family and friends
- You should have a specific body size, and you should spend your compensation money to sustain your body
- You should avoid having problems with trackers
- You have to own a phone or find an alternative way of communicating with trackers
- You should not complain about trackers and research teams because they are taking care of you
- In order to graduate from the trial you have to promote per-protocol compliance, and only then might you be invited to other projects

Many international projects compete with each other for funds, projects, staff, and participants, and both staff and volunteers act properly in order to produce fine-looking publications and presentations that allow further expansion of research “archipelagos” (Geissler, 2013). In this dynamic interchange, a good participant delivers adherence and compliance. A good researcher or implementer commodifies participation and gets new projects, offering subsequent participation to proven good participants. By following etiquette rules, participants provide research teams with quality scientific data, thus allowing projects to expand, which in turn creates employment for researchers. These various efforts to keep afloat a mobile sovereign such as “Little America” and remain linked up with it are enacted through etiquette. All actors in the trials jointly create an economy of good behaviours in which the healthy body becomes both a source of commodification and an occasion for a subject to invest in themselves. This allows both researchers and participants to co-opt and commodify biovalue, and gives them a reason to “stick together” at the research site.

## Good trial subjects, bad collaborators

To theorize a relationship between bioetiquette and the making of compliant trial subjects, it might be useful to engage with Hacking’s (2007) idea of “looping effects”. Hacking shows that stereotypes are produced within social contexts – institutions and their expert knowledge “loop” people into groups, creating “kinds” of humans – and that such stereotyping changes how people subjected to classification understand themselves (Hacking, 2006, 2007). Anthropologists have used the notion of looping effects to describe the making of new human entities by global health institutions, such as “gay men”, “people-at-risk”, and “collaborators” (Holt, 2013; Lock & Nguyen, 2010; Moyer & Nguyen, 2015).

The making of “trial subjects” in this trial is an example of a powerful looping effect, where social networks reinforced and repeated acts of stereotyping. Using Hacking’s elegant framework, we can see four elements: (1) people from local precarious communities (2) voluntarily embraced a locally new category of “trial subject” and attended various study and follow-up visits, allowing (3) expert researchers to validate compliant participants as “human subjects” and in an ethically sound way extract biovalue from them, while commodifying their per-protocol compliant data, and, in return, provide (4) “good” trial subjects with benefits, maintaining and legitimizing their classification as “trial subjects”. It is crucial to this looping effect that the relations between experts and trial subjects were shaped by unwritten rules of etiquette – that is, by half-hidden knowledge about how people ought to behave in order to be classified as “proper” participants.

Bioetiquette is not limited to shaping the behaviours of trial subjects on the site where I conducted my research. Archipelagos like Little America are in a highly competitive market for funding and projects, and international reputation is directly linked both to a continuous stream of successive grants, and the employment of many people. After presenting my fieldwork findings at an international bioethics conference in 2017, I received several emails from representatives of Little America who expressed concern that I was a “bad collaborator”. I was admonished for disrespecting internal rules and procedures, and asked to sign various institutional agreements and submit my writings to the internal publishing committee. Despite a lot of moral pressure, I was able to decline this request: I did, after all, obtain all the necessary permissions for my research from the local ethical committee and the ethical committee of my home institution. However, I did feel embarrassed and ashamed that I was considered an “improper” and biased collaborator. In analysing this situation and my response, I came to realize that I, too, was targeted by a looping effect and yet another kind of bioetiquette: a powerful institution had produced expert knowledge about me, I was stereotyped as a bad collaborator, and due to my research efforts this category got coined and gained salience.

## Bioetiquette as a collateral product of formal bioethics

There is a growing concern that normative bioethics tends to simplify the contexts and processes in which bioethics is practised (Elliott, 1999; Marshall, 1992). Salter and Jones (2005) have powerfully shown that bioethics acts like a science-based technocratic model that produces various epistemological accounts on behalf of a new kind of authority. Rabinow and Rose (2003) highlight that bioethics enacts a “complex” that exercises biopolitics. In a similar manner, Zylinska (2009) argues that bioethics is increasingly becoming a set of regulations and forces for effective commodification and life management.

Adding to critical scholars who exposed the “grey zones” of bioethics in real world practices, I suggest that it is time to also analyse and discuss bioetiquette. Originally this term had nothing to do with the half-hidden rules for the proper behaviour of clinical labourers. When over two decades ago Martin and Stent (1998) coined “bioetiquette” as a counterpart to bioethics, they took it to be a solely positive phenomenon that should help govern civility in biomedical research through proposed rules of noble behaviour for scientists. Inspired by my informants and ideas from science and technology studies (STS) – especially that science is permeated by wider sociocultural, political, and economic forces (Latour & Woolgar, 1986) – I seek to broaden the original meaning of the term bioetiquette. In an era when labour markets promote the self-responsibilization of workers in semi-regulated and flexible settings (Scully, 2016), I suggest that bioetiquette pertains to the behavioural adjustments taken on board by people who are willing to be affiliated with powerful biomedical organizations, such as trial volunteers, security guards, collaborators, etc. Bioetiquette does not just involve people, however; non-human actors also support proper behaviours. ID cards, graduation ceremonies and certificates, appointment cards, bureaucracies and protocol compliance, social emotions (trust, loyalty, honour, embarrassment or guilt), and shame and repulsion against “myths”, “non-compliance” and “misconceptions” all play their part in enticing participants into compliance. As much as bioethics is inseparable from biopower, biopolitics, and globalizing aid governance, bioetiquette is also inseparable from bioethics and other systems of rules that allow individuals and communities to accept painful or difficult measures with the assumption that this is for their own good. Moreover, bioetiquette is likely to be co-modulated by other contextual forms of etiquette––e.g. communication etiquette, hospital etiquette, gender-related etiquette––in any given setting.

The key analytic point emerging from the case presented here is that bioetiquette is not an exceptional phenomenon of Little America, but an ordinary process related to the harmonization of ethics, clinical research regulation, and conduct. According to the website https://clinicaltrials.gov, in 2019 there were 297,685 clinical studies approved in the world (National Institute of Health, 2018). The majority of them, if not all, involve ethical expertise, surveillance, and evaluation in accordance with major international guidelines such as “Good Clinical Practice” (GCP), a forty-nine-page document consisting of standardized operational procedures outlining what good researchers, ethical boards, sponsors, and trial subjects should do. It is slowly but surely replacing the quest for decency that inspired the Helsinki Declaration with rather dry, technical, and detached sets of regulations (Kimmelman, Weijer, & Meslin, 2009). In the era of standardized research, the GCP is a key source that is replicated in various settings, perhaps making it what Rottenburg (2005) calls a “meta-code” that can be reproduced and tailored to the context. This line of reasoning places the matrix of the meta-code outside of the scope of criticism and suggests that all that can be problematized is the implementation: cultures, people, their momentums and their knowledge. Bioetiquette, then, is a force that helps with the repetition of the meta-code of formal bioethics in concrete settings at this or that specific moment.

Whilst bioethics generates judgements on what are good and bad research protocols, these protocols themselves, by stealth, prescribe how to properly behave to meet the required conditions. In this way, bioetiquette safeguards the quality, breadth and consistency of scientific data obtained from trial subjects in clinical research. It stimulates good per-protocol compliance. And while a vast bureaucracy imposes ethics rules on research practices, it is the etiquette that regulates the behaviour of all those involved. With a growing realization that what counts for most might not be good intentions but their real world effects, bioetiquette may prove to be a missing term alongside biocapital, bioeconomy, biovalue and biopower––which all unfold around clinical research praxis.

## Where to go from here

In 1982, Toulmin famously argued that medicine saved the life of ethics as it carried with it the suggestions that doctors routinely judge what is good and bad for people. Nearly forty years later it is clear that ethics, once saved, became bioethics: authoritative institutionalized norms for what is good and bad in clinical research. Bioethics has contributed to the establishment of various institutions, including ethical boards, contract research organizations, research units, university departments, and their attendant bureaucracies. Bioethics has introduced new kinds of people, provided authoritative knowledge about them, and delivered an infrastructure to inspect those entities. By producing authoritative accounts on how to behave and manage social interactions in research professionally and smoothly, bioethics, in return, is generating, as a side effect, a bioetiquette unique to each research context.

This article presents a snapshot of the bioetiquette in a specific time and in the context of Ebola vaccine research in West Africa. This particular case cannot be generalized, but it does suggest that there is a need for more research into bioetiquette, and into the relationship between bioetiquette and bioethics in other clinical trials and other kinds of biomedical interventions. For it is a problem to just study, issue diplomas for, and read and write about ethics, while ignoring the etiquette which is so prominent on the ground.

## Note

1 Here, as throughout the text, if not otherwise specified it is the informant's own emphasis that has been added.
